# Stretcher: a learning-based framework for deformation-robust keypoint descriptors

**DOI:** 10.1007/s11548-026-03698-w

**Published:** 2026-05-27

**Authors:** Constantin von Witzleben, Nazim Haouchine

**Affiliations:** 1 Brigham and Women’s Hospital, Harvard Medical School, Boston, USA; 2 Technical University of Munich, Munich, Germany

**Keywords:** Surgical tracking, Image registration, Keypoint matching, Deformation robustness, Image features

## Abstract

**Purpose:**

Keypoint-based tracking and navigation are central to many image-guided surgical systems, yet their reliability degrades significantly in the presence of soft-tissue deformation, as commonly observed in procedures such as liver surgery. Existing keypoint descriptors often fail to remain stable under large affine and non-rigid transformations. This work introduces Stretcher, a framework designed to improve the deformation robustness of keypoint descriptors for surgical tracking and navigation.

**Methods:**

Instead of directly matching keypoint descriptors across deformed images, Stretcher learns a neural model that explicitly captures the effect of affine deformations on descriptor representations. At inference time, a grid of affine transformations is simulated, and keypoint descriptors are efficiently adjusted using the learned model, avoiding redundant descriptor recomputation. This enables robust descriptor adaptation under deformation while maintaining computational efficiency.

**Results:**

Stretcher is evaluated on liver surgery images exhibiting large tissue deformation. Experimental results show that the proposed approach improves keypoint matching robustness in highly deformed regions. Across all evaluated scenarios, Stretcher maintains state-of-the-art precision while providing higher matching accuracy compared to existing descriptor-based methods, particularly where deformation is severe.

**Conclusion:**

By explicitly modeling the impact of affine deformations on keypoint descriptors, Stretcher enables reliable keypoint matching under complex tissue motion. The proposed framework improves robustness in challenging surgical scenarios without sacrificing efficiency, making it well suited for deformation-prone intraoperative tracking and navigation tasks. Code is available at https://github.com/constiwitzleben/Stretchers.

## Introduction

Keypoint detectors, descriptors, and matchers are core components of many computer vision pipelines, enabling reliable correspondences for tasks, such as image registration, tracking, and 3D reconstruction [1–3]. In medical imaging and image-guided surgery, accurate keypoint matching is critical for applications including surgical navigation and intraoperative tracking. However, soft-tissue deformation caused by stretching, cutting, and tool interaction frequently violates the assumptions of rigid or globally affine motion, leading to rapid degradation in descriptor reliability [4–6].

**Fig. 1:**
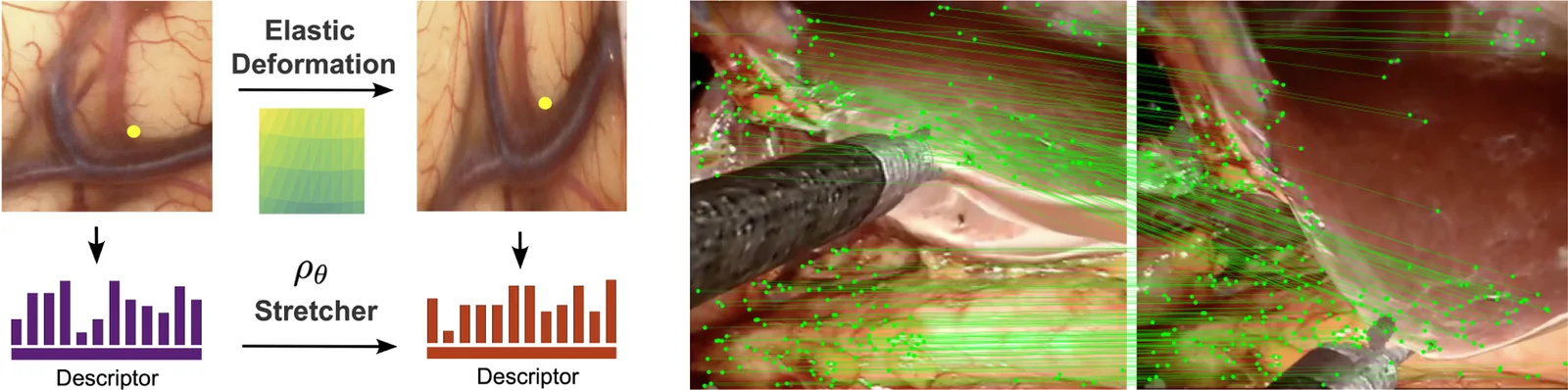
Visualization of the Stretcher framework. We aim to learn to transform a keypoint descriptor in latent space rather than image space to improve its matching potential under elastic deformation, as seen on the left image. On the right image, is a use case for Stretcher that can be used with surgical data (From the DejaVu dataset [14])

Classical descriptors such as SIFT offer invariance to rotation and scale but perform poorly under non-rigid deformation [7]. Learning-based approaches, including SuperPoint, DISK, and ALIKED, improve robustness through data-driven training and augmentation [8–10], yet remain brittle when deformation is strong, localized, and non-uniform, as commonly observed in surgical scenes. Test-time augmentation can partially address this limitation but requires repeated feature extraction and is often computationally prohibitive for real-time use. More recently, steerable descriptor frameworks have demonstrated efficient invariance to rotation and affine transformations by learning descriptor-space mappings at inference time [11, 12], but they do not explicitly target deformation patterns arising from soft-tissue mechanics.

In this work, we introduce *Stretcher*, a learning-based framework that improves keypoint matching robustness under localized tissue deformation by transforming descriptors directly in latent space. Stretcher learns how keypoint descriptors change under local affine strain, which serves as a first-order approximation of non-rigid deformation, and generates multiple deformation-conditioned descriptor hypotheses per keypoint without recomputing image features. This design enables deformation-aware matching while remaining fully compatible with existing matchers such as Dual Softmax and LightGlue [13]. Stretcher is trained on real surgical images with synthetically applied affine deformations to provide explicit supervision of descriptor transformations while avoiding the need for dense ground-truth correspondences in real deformed tissue. Experimental results on synthetically deformed endoscopic images and real surgical data demonstrate improved matching reliability in highly deformed regions compared to standard pipelines.

## Methods

Stretcher aims to improve keypoint matching robustness under localized tissue deformation by learning how keypoint descriptors change under deformation and applying this transformation directly in latent space. An overview of the framework is shown in Fig. 1. In the following, we first describe the general image registration setting and the role of test-time augmentation for handling deformation. We then introduce the Stretcher framework, detailing the deformation model, network architecture, and loss function. Finally, we describe the training strategy and analyze the performance of the learned affine descriptor transformations.

### Image registration with test-time augmentation

We consider the problem of registering two images of the same scene acquired at different time points, referred to as a rest image $\textbf{I}^r$ and a deformed image $\textbf{I}^d$. Between the two time points, the scene undergoes an unknown transformation *t* such that $t(\textbf{I}^r) = \textbf{I}^d$. Keypoints $\textbf{k}^r$ and $\textbf{k}^d$ are detected independently in both images, and local descriptors $\textbf{d}^r$ and $\textbf{d}^d$ are extracted using a standard backbone.

A common strategy to improve robustness under deformation is test-time augmentation. In this framework, multiple candidate transformations $t'_k$ are applied to the rest image and descriptors are recomputed for each transformed instance. During matching, all resulting rest image descriptors $\textbf{d}^{r^k}$ are considered and matched with the deformed image descriptors $\textbf{d}^d$. Correspondences are established by selecting the best-scoring matches across the set of candidate transformations. For example, in Dual Softmax matching, the matching matrix is computed as $M_{ij} = \max \limits _k{d_i^{r^k}}^\top d_j^d$. If one of the candidate transformations provides a good approximation of the true underlying deformation, the corresponding set of rest image descriptors will be more similar to the deformed image descriptors and more matches can be found.

While effective, test-time augmentation is computationally expensive, as it requires repeated image deformation, feature extraction, and descriptor computation for each candidate transformation. This cost grows rapidly with the number of deformation hypotheses considered and can be prohibitive for real-time surgical applications, motivating more efficient approaches that retain deformation-aware matching without repeated inference.

### Stretcher: descriptor-space deformation modeling

Stretcher removes the need for repeated inference in test-time augmentation by learning how keypoint descriptors change under deformation and applying this transformation directly in latent space. Rather than deforming images and recomputing features for each deformation hypothesis, Stretcher generates deformation-conditioned descriptor hypotheses from a single descriptor extracted at the rest image.

#### Local deformation parameterization

We model local tissue deformation using an affine strain representation parameterized by horizontal strain $\sigma _x$, vertical strain $\sigma _y$, and shear strain $\sigma _{xy}$. Although surgical tissue motion is nonlinear and spatially varying, affine transformations provide a first-order local approximation that captures dominant deformation modes while remaining computationally efficient and easy to parametrize. By evaluating multiple local affine hypotheses, complex non-rigid behavior can be approximated through a combination of local linear models.

To improve robustness to rotational artifacts under large deformations, we adopt a co-rotated strain formulation inspired by finite element modeling. Given a deformation gradient $\textbf{F}$ constructed from the affine parameters, we perform a QR decomposition $\textbf{F} = \textbf{RS}$, where $\textbf{R}$ represents rigid-body rotation and $\textbf{S}$ encodes pure stretch and shear. The co-rotated strain $\textbf{E}_c = \mathbf {S - I}$ isolates deformation independently of rotation and improves numerical stability under large local deformations. The parameters $\sigma _x$, $\sigma _y$, and $\sigma _{xy}$ are then derived from $\textbf{E}_c$.

#### Descriptor transformation network

Given a keypoint descriptor $\textbf{d}^r$ extracted from the rest image and the corresponding strain parameters $\alpha = \{\sigma _x, \sigma _y, \sigma _{xy}\}$, Stretcher learns a deformation-aware mapping ρ that predicts the descriptor appearance under deformation. The network consists of three lightweight multi-layer perceptrons (MLPs), each modeling the effect of one strain component. Their outputs are summed and added to the original descriptor

1 $$\begin{aligned} \rho (\alpha ,\textbf{d}) = \textbf{d} + \sum _{i=1}^{3} \textrm{MLP}_i(\alpha , \textbf{d}). \end{aligned}$$

This residual formulation encourages the network to preserve stable descriptors under small deformations while selectively adapting features for more significant strain.

#### Loss function

Given rest descriptors $\textbf{d}^r$ and the corresponding deformed descriptor $\textbf{d}^d$, Stretcher is trained to predict $\textbf{d}^d$ from $\textbf{d}^r$ and the associated strain parameters α. We use a mean squared error loss between predicted and target descriptors

2 $$\begin{aligned} L = \frac{1}{N}\sum _{i=1}^{N} \left( \rho (\alpha _i,d^r_i) - d^d_i\right) ^2 \end{aligned}$$

where *N* denotes the number of training descriptor pairs. This objective directly supervises the learned descriptor transformation while leaving the underlying keypoint detector and matcher unchanged.

**Fig. 2:**
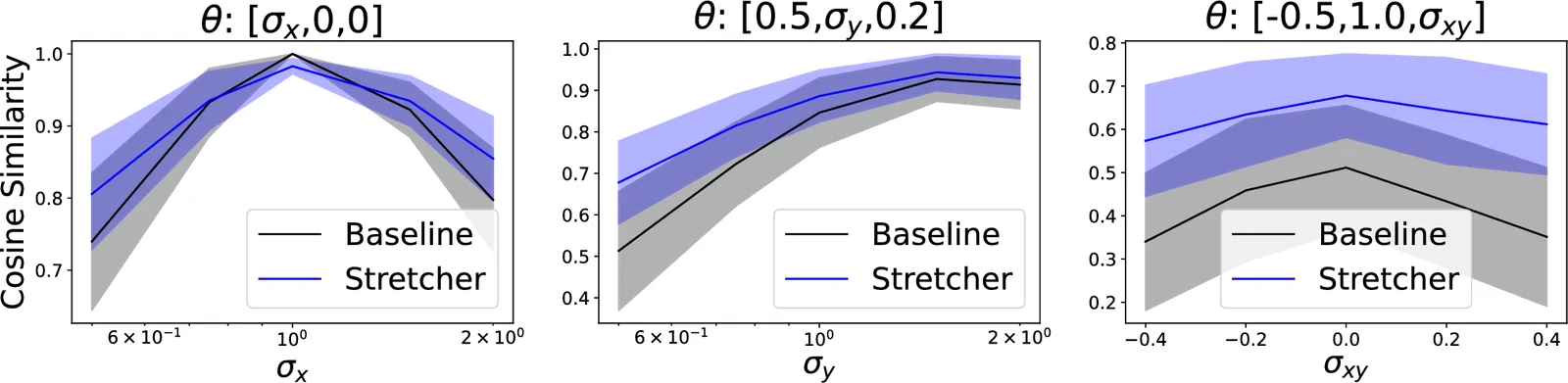
Stretcher improves for larger affine transformation parameters while showing minimal improvements for smaller ones

**Fig. 3:**
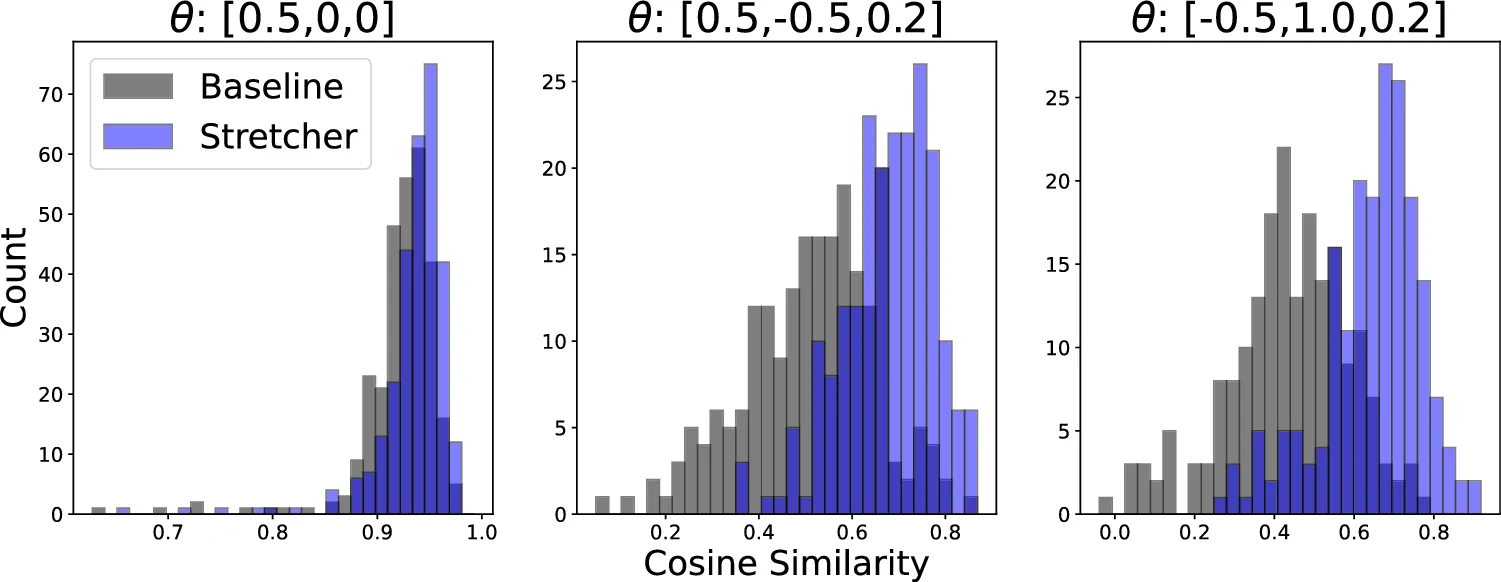
Histogram of cosine similarity between keypoint descriptions and their corresponding deformed descriptions. Baseline shows similarities before and Stretcher shows similarities after applying Stretcher with known affine parameters

### Training stretcher

Stretcher can be applied to any keypoint descriptor and trained on arbitrary image data. In this section, we outline the specific design choices made for the present implementation and analyze the behavior of the learned descriptor transformation under affine deformation.

#### Data and implementation choices

We train on images from the Cholec80 dataset [15], excluding hepatic surgery sequences from training and reserving them exclusively for evaluation. Cholec80 provides a large and diverse collection of real laparoscopic images representative of intraoperative conditions, making it well suited for learning descriptor transformations in a surgical context. To generate supervised training pairs, we apply controlled affine deformations to real surgical images, allowing exact correspondence between keypoints in the rest image and their locations in the deformed image. This strategy is necessary because learning a descriptor transformation requires access to ground-truth deformed descriptors at corresponding keypoint locations, which are generally unavailable in real surgical data due to the lack of dense and reliable expert annotations under tissue deformation.

We use SuperPoint [8] as the descriptor backbone in this work. SuperPoint is a recent and widely adopted keypoint descriptor that has demonstrated strong performance across a range of vision tasks, including medical and surgical applications. Importantly for our setting, SuperPoint provides dense feature maps that can be interpolated at arbitrary keypoint locations, enabling precise extraction of corresponding descriptors before and after deformation.

The strain parameters $\sigma _x$ and $\sigma _y$ are discretized into $\{-1, -0.5, 0, 0.5, 1\}$, while $\sigma _{xy}$ $\sigma _{xy}$ is discretized into $\{-0.4, -0.2, 0, 0.2, 0.4\}$. This results in $5^3 = 125$ candidate local affine deformation modes. During training, a subset of these deformation hypotheses is randomly sampled per image to generate paired descriptors $(\textbf{d}^r, \textbf{d}^d)$ with known correspondence. Using these parameters we generated 137 200 training descriptor pairs. Training Stretcher on them took approximately 30 min.

**Table 1:** Mean ± std. for key metrics across SOTA keypoint matchers compared to Stretcher, grouped by matching method

Matcher	Method	Prec. (%)	Match Sc. (%)	# Matches	Entropy	SBP
DSM	DISK	24.84 ± 25.08	0.31 ± 0.31	16.25 ± 9.68	0.16 ± 0.17	0.09 ± 0.09
DSM	ALIKED	25.57 ± 23.78	**17**.**66** ± **17**.**03**	359.25 ± 55.03	0.60 ± 0.12	0.20 ± 0.18
DSM	SP	28.99 ± 13.11	12.90 ± 6.58	173.5 ± 31.57	**0**.**87** ± **0**.**05**	0.27 ± 0.12
DSM	**Stretcher**	**43**.**10** ± **16**.**99**	12.83 ± 5.55	117.0 ± 17.07	0.84 ± 0.08	**0**.**40** ± **0**.**17**
LG	DISK	71.07 ± 41.08	28.50 ± 19.64	614.0 ± 417.32	0.58 ± 0.34	0.59 ± 0.36
LG	ALIKED	**94**.**75** ± **5**.**95**	**62**.**29** ± **20**.**08**	405.0 ± 108.60	0.88 ± 0.04	**0**.**88** ± **0**.**12**
LG	SP	85.92 ± 10.70	23.71 ± 13.58	111.25 ± 67.05	0.80 ± 0.16	0.73 ± 0.23
LG	**Stretcher**	75.25 ± 11.78	−	200.0 ± 0.0	**0**.**91** ± **0**.**05**	0.71 ± 0.15

The entropy is presented in the local strain magnitudes at the locations of correct matches. SBP is the strain-balanced precision and averages the precision over discrete strain levels. Both should be large for effective matching under deformation

Bold signifies the best score per metric

#### Analysis of learned descriptor transformations

To investigate how well Stretcher models affine deformation in descriptor space, we analyze the cosine similarity between rest and deformed descriptors before and after applying the learned transformation. Figure 2 shows descriptor similarity as a function of individual affine strain components. Stretcher significantly increases similarity by up to 0.3 for larger deformations, while introducing minimal changes for small strains, indicating that the learned transformation selectively compensates for geometric distortion rather than uniformly altering descriptors.

**Fig. 4:**
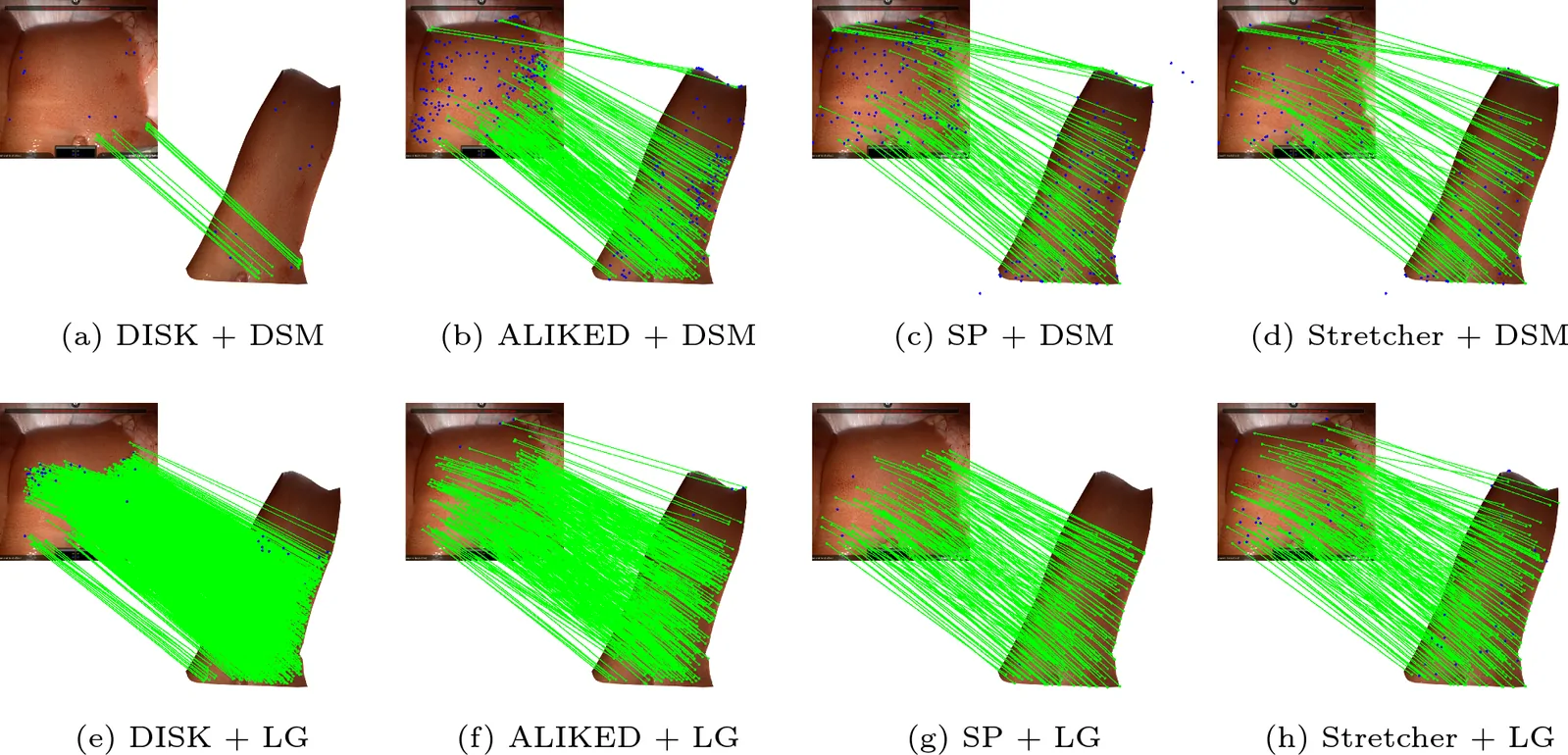
Visualization of the matches found for a synthetically deformed liver from a surgical image. Correct matches are shown in green and incorrect matches with blue points. Stretcher provides a more even distribution of points, in particular in highly-stressed regions

**Fig. 5:**
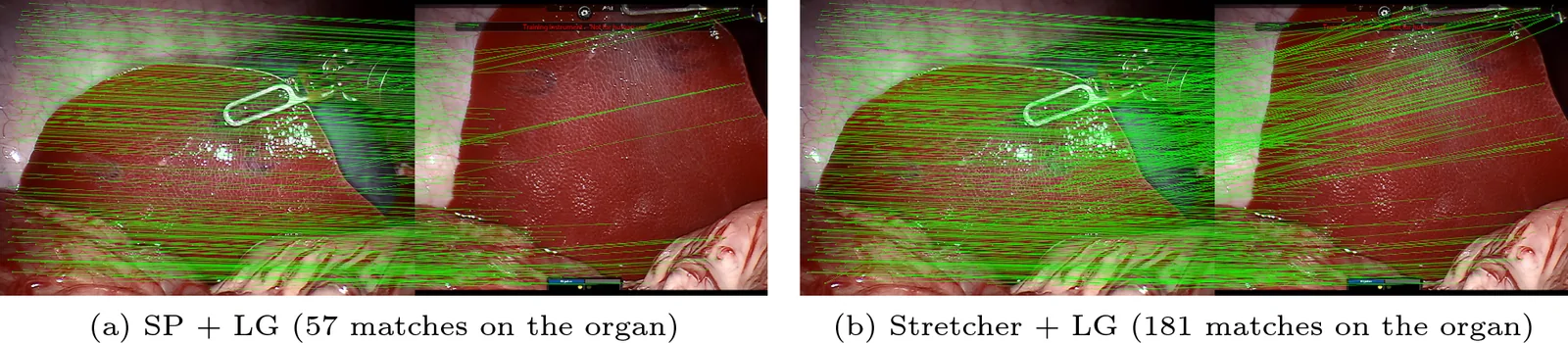
Matched keypoints for a real deformed pig liver. The SuperPoint and LightGlue example on the left has 262 matches of which 57 are on the liver. The Stretcher example on the right has 500 matches of which 181 are on the liver (3× more)

Figure 3 further illustrates the distribution of cosine similarities across different deformation modes. The results confirm that Stretcher improves descriptor alignment under increasing deformation magnitude, which can enable more downstream matches for images pairs with significant deformations.

Importantly, both analyses are conducted on held-out test. The consistent improvement in descriptor similarity across unseen descriptor pairs indicates that Stretcher generalizes beyond the specific training instances and learns a transferable mapping between local affine deformation and its effect in descriptor space, rather than memorizing individual training examples.

## Results

### Performance and comparison with similar methods

We evaluate Stretcher on real surgical images with both synthetic FEM- and real surgical deformations. Synthetic deformations enable quantitative evaluation using known ground-truth keypoint correspondences which are generally unavailable for real surgical deformations. Real surgical data allows us to validate the behavior of the method under realistic intraoperative conditions.

### Results on synthetically deformed surgical images

Table 1 compares Stretcher with the state-of-the-art keypoint matching pipelines. We include DISK, ALIKED, and SuperPoint as image keypoint detectors and descriptors and use Dual Softmax (DSM) and LightGlue (LG) for matching. We report the precision, match score and number of matches as classical image matching metrics. In addition, we report the entropy of local strain magnitudes at correct match locations and the strain-balanced precision (SBP). SBP averages precision across discrete strain levels and reflects matching performance under varying deformation severity. High scores indicate that correct matches are evenly distributed across regions with different deformation magnitudes.

Under Dual Softmax, Stretcher achieves the highest precision and SBP, indicating a strong ability to preserve reliable correspondences under deformation. Although the total number of matches is lower than some baselines, Stretcher prioritizes matches in highly stressed regions where standard methods frequently fail. Under LightGlue, Stretcher maintains competitive precision while achieving the highest entropy in strain magnitudes at correct match locations, indicating a more spatially balanced distribution of matches across deformation levels.

Using Dual Softmax and SuperPoint, standard matching takes 1.4 s, Stretcher takes 6.1 s and standard test-time augmentation takes 160 s. For LighGlue, Stretcher is not yet optimized.

Figure 4 visualizes matching results on a synthetically deformed liver image, where green connections denote correct matches and blue markers indicate incorrect correspondences. Compared to baseline pipelines, Stretcher produces less dense but more evenly distributed correct matches, particularly in regions undergoing strong elastic deformation. In contrast, baseline methods tend to concentrate matches in weakly deformed areas while producing few or incorrect matches in high-strain regions. This qualitative behavior is consistent with the quantitative strain-based metrics reported above and is desirable for surgical navigation tasks, where deformation is often localized and non-uniform. Importantly, such highly deformed regions frequently correspond to areas of surgical interaction, for example where instruments contact tissue or where cutting and manipulation occur, making robust correspondences in these regions particularly relevant.

### Results on real surgical deformations

To complement the synthetic evaluation, we assess Stretcher’s performance on real surgical image pairs of a deformed pig liver. While dense ground-truth correspondences are unavailable, we analyze both the number of matches and their spatial distribution. Figure 5 shows example matching results on a real surgically deformed image pair. The liver is manually segmented to identify keypoints located on the organ surface. Using the baseline SuperPoint + LightGlue pipeline, 262 matches are obtained, of which only 57 lie on the liver and are sparsely distributed. In contrast, Stretcher produces 500 matches, with 181 correspondences located on the liver and more evenly distributed across the organ surface.

Taken together, these results suggest that within a test-time augmentation framework, local first-order affine deformation hypotheses can collectively model the dominant effects of non-rigid surgical tissue motion. Moreover, the Stretcher network successfully captures the effect of such deformations in descriptor space, enabling improved matching performance in real deformed surgical scenarios.

## Conclusion and discussion

We presented Stretcher, a learning-based framework for improving keypoint matching robustness under localized surgical tissue deformation by transforming descriptors directly in latent space. By modeling the effect of local affine deformation on descriptor representations, Stretcher enables deformation-aware matching without repeated feature extraction and remains fully compatible with existing matching pipelines.

Experimental results on synthetically deformed surgical images demonstrate improved matching reliability in highly deformed regions, while evaluations on real surgically deformed liver images show consistent gains in both the number and spatial distribution of matches. These findings indicate that local first-order affine approximations, when combined within a test-time augmentation framework, can effectively capture dominant aspects of non-rigid surgical deformation in descriptor space.

Future work will focus on validating Stretcher on larger and more diverse sets of real surgical data and exploring richer deformation models to further improve robustness in complex intraoperative scenarios.
